# Supplementary figures and images for: Genomic characterization and phylogenetic analysis of the first SARS-CoV-2 variants introduced in Lebanon
Source: PeerJ. 2021 Mar 16;9:e11015. doi: 10.7717/peerj.11015 (PMC8447710; doi:10.7717/peerj.11015)

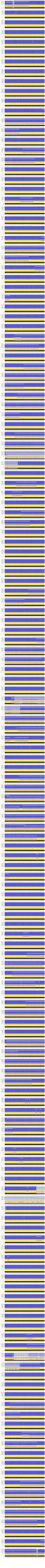

Supplement: Supplemental Information 1 [file peerj-09-11015-s001.pdf]
