# Supplementary material for: Genomic characterization and phylogenetic analysis of the first SARS-CoV-2 variants introduced in Lebanon
Source: PeerJ. 2021 Mar 16;9:e11015. doi: 10.7717/peerj.11015 (PMC8447710; doi:10.7717/peerj.11015)
Supplement: Supplemental Information 2 — Metadata table includes isolate name, accession number, collection date, location, travel history, and lineage information. [file peerj-09-11015-s002.pdf]

| Isolate_name | Accession_number | Collection_Date | Location | Travel_History | Lineage |
|---|---|---|---|---|---|
| HGRC-2-2162 | EPI_ISL_437512 | 2020-03-26 | Iran | Unknown | B.4 |
| KHGRC-2-2162 | EPI_ISL_442044 | 2020-03-26 | Iran | Unknown | B.4 |
| KHGRC-1.1-IPI-8206 | EPI_ISL_442523 | 2020-03-09 | Iran | Unknown | B.4 |
| BC_37_0-2 | EPI_ISL_412965 | 2020-02-16 | Canada | Iran | B.4 |
| L2409 | EPI_ISL_435131 | 2020-02-25 | UAE | Unknown | B.4 |
| HKU-903b | EPI_ISL_434566 | 2020-01-27 | Hong_Kong | Unknown | B.4 |
| L6627 | EPI_ISL_435139 | 2020-02-25 | UAE | Unknown | B.4 |
| IVDC-SD-001 | EPI_ISL_408482 | 2020-01-19 | China | Unknown | B |
| SH0126 | EPI_ISL_416407 | 2020-02-15 | China | Unknown | B.4 |
| SH0070 | EPI_ISL_416373 | 2020-02-07 | China | Unknown | B.4 |
| HBCDC-HB-05 | EPI_ISL_412981 | 2020-01-18 | China | Unknown | B.4 |
| JX124 | EPI_ISL_421260 | 2020-01-26 | China | Unknown | B.4 |
| SH0022 | EPI_ISL_416331 | 2020-01-30 | China | Unknown | B.4 |
| Bangkok-0078 | EPI_ISL_447019 | 2020-04-01 | Thailand | Unknown | B.4 |
| LA-NCDC-01760 | EPI_ISL_435106 | 2020-03-18 | India | Unknown | B.4 |
| KU12 | EPI_ISL_416458 | 2020-03-02 | Kuwait | Iran | B.4 |
| UN-1115 | EPI_ISL_421667 | 2020-03-10 | India | Iran | B.4 |
| LA-NCDC-01441 | EPI_ISL_435101 | 2020-03-15 | India | Unknown | B.4 |
| Norway_1380 | EPI_ISL_417484 | 2020-02-26 | Norway | Iran | B.4 |
| Tb-82 | EPI_ISL_415644 | 2020-02-28 | Georgia | Unknown | B.4 |
| HSGM-8992 | EPI_ISL_429865 | 2020-03-18 | Turkey | Unknown | B.4 |
| HSGM-1468 | EPI_ISL_437312 | 2020-03-25 | Turkey | Unknown | B.4 |
| KU09 | EPI_ISL_416541 | 2020-03-02 | Kuwait | Unknown | B.4 |
| CAMB-78BB7 | EPI_ISL_441184 | 2020-04-03 | England | Unknown | B.1.1 |
| PHWC-2E01B | EPI_ISL_446047 | 2020-04-10 | Wales | Unknown | B.1.1 |
| OXON-ADFF9 | EPI_ISL_448654 | 2020 | England | Unknown | B.1.1 |
| WA-S720 | EPI_ISL_449890 | 2020-04-12 | USA | Unknown | B.1.1 |
| StPetersburg-RII7039V | EPI_ISL_450247 | 2020-04-16 | Russia | Unknown | B.1.1 |
| OXON-AF09A | EPI_ISL_448808 | 2020-04-07 | England | Unknown | B.1.1 |
| DHWM-03041 | EPI_ISL_417422 | 2020-03-04 | Belgium | Unknown | B.1.1 |
| ChVir2225 | EPI_ISL_450496 | 2020-03 | Lithuania | Unknown | B.1.1 |
| OXON-AEFF8 | EPI_ISL_448804 | 2020-04-07 | England | Unknown | B.1.1 |

| Isolate_name | Accession_number | Collection_Date | Location | Travel_History | Lineage |
|---|---|---|---|---|---|
| LOM-INMI-13075-N | EPI_ISL_451309 | 2020-03-01 | Italy | Unknown | B.1 |
| NOR-N4427 | EPI_ISL_428367 | 2020-03-23 | France | Unknown | B.1 |
| Poland_1104795 | EPI_ISL_428232 | 2020-03-18 | Poland | Unknown | B.1 |
| UGent-64 | EPI_ISL_451175 | 2020-04-07 | Belgium | Unknown | B.1 |
| PA-MGSC18-04 | EPI_ISL_436473 | 2020-03-25 | USA | Unknown | B.1 |
| ARA-10552 | EPI_ISL_419168 | 2020-03-17 | France | Unknown | B.1 |
| NRC-01 | EPI_ISL_430820 | 2020-03-18 | Egypt | Unknown | B.1 |
| KAUST-Madinah272 | EPI_ISL_437753 | 2020-03-30 | KSA | Unknown | B.1 |
| CT-UW272 | EPI_ISL_418057 | 2020-03-13 | USA | Unknown | B.1 |
| WA-UW-1420 | EPI_ISL_423000 | 2020-03-18 | USA | Unknown | B.1 |
| OH_0019 | EPI_ISL_426430 | 2020-03-08 | USA | Unknown | B.1.255 |
| NTU08 | EPI_ISL_422411 | 2020-03-14 | Taiwan | Egypt | B.1 |
| ON-PHL-8751 | EPI_ISL_418345 | 2020-02 | Canada | Unknown | B.1 |
| KAUST-Makkah163 | EPI_ISL_437697 | 2020-04-06 | KSA | Unknown | B.1 |
| PA-CDC-2908 | EPI_ISL_447844 | 2020-03-08 | USA | Unknown | B.1 |
| CGMH-CGU-23 | EPI_ISL_444276 | 2020-03-21 | Taiwan | Unknown | B.1 |
| Qc-L00241292 | EPI_ISL_450316 | 2020-03-27 | Canada | Unknown | B.1.255 |
| NRC-03 | EPI_ISL_430819 | 2020-03-18 | Egypt | Unknown | B.1.246 |
| AR-ISPCH-14 | EPI_ISL_445348 | 2020-03-25 | Chile | Unknown | B.1 |
| CA-SCCPHD-UC104 | EPI_ISL_435583 | 2020-02-29 | USA | Unknown | B.1 |
| MAG-GVI-93457 | EPI_ISL_447778 | 2020-04-01 | Colombia | Unknown | B.1.255 |
| Finland_13M82 | EPI_ISL_418399 | 2020-03-13 | Finland | Unknown | B.1 |
